# RNA-Targeted Therapies and High-Throughput Screening Methods

**DOI:** 10.3390/ijms21082996

**Published:** 2020-04-23

**Authors:** Siran Zhu, Saul Rooney, Gracjan Michlewski

**Affiliations:** 1 Infection Medicine, University of Edinburgh, The Chancellor’s Building, 49 Little France Crescent, Edinburgh EH16 4SB, UK; 2 Zhejiang University-University of Edinburgh Institute, School of Medicine, Zhejiang University, 718 East Haizhou Rd., Haining 314400, China

**Keywords:** RNA, microRNA, RNA-binding proteins, small molecules

## Abstract

RNA-binding proteins (RBPs) are involved in regulating all aspects of RNA metabolism, including processing, transport, translation, and degradation. Dysregulation of RNA metabolism is linked to a plethora of diseases, such as cancer, neurodegenerative diseases, and neuromuscular disorders. Recent years have seen a dramatic shift in the knowledge base, with RNA increasingly being recognised as an attractive target for precision medicine therapies. In this article, we are going to review current RNA-targeted therapies. Furthermore, we will scrutinise a range of drug discoveries targeting protein-RNA interactions. In particular, we will focus on the interplay between Lin28 and let-7, splicing regulatory proteins and survival motor neuron (SMN) pre-mRNA, as well as HuR, Musashi, proteins and their RNA targets. We will highlight the mechanisms RBPs utilise to modulate RNA metabolism and discuss current high-throughput screening strategies. This review provides evidence that we are entering a new era of RNA-targeted medicine.

## 1. Introduction

Eukaryotic post-transcriptional regulation is a critical process that controls RNA metabolism and gene expression. RNA-binding proteins (RBPs) are key regulators of post-transcriptional events such as RNA maturation, localisation, modification, silencing, translation, and turnover [1]. This is achieved by recognising and binding specific RNA motifs or structures. So far, more than 1000 RBPs have been identified in humans. Most of them are ubiquitously expressed, with only a small percentage showing a tissue-specific expression pattern [2].

Mutations that affect post-transcriptional events often lead to serious diseases [3]. Hence, RNA-targeted therapeutic strategies have shown promising results by correcting aberrant activities of RNA metabolism and rescuing pathological phenotypes [4,5]. The most common approaches of RNA-targeted therapies include RNA interference (RNAi) using small interfering RNAs (siRNAs), microRNAs or ribozymes, modulating RNA-protein interactions, and RNA–RNA interactions using chemically modified antisense oligonucleotides (ASOs) or small molecules. Besides these therapies, mRNA delivery therapy is aimed at directly enhancing gene expression [6]. The first RNA-targeted therapy, fomivirsen, was approved by the Food and Drug Administration (FDA) in 1998. Fomivirsen is an ASO drug for the treatment of cytomegalovirus (CMV) retinitis in immunocompromised patients. The drug acts by targeting the mRNA of the CMV immediate-early-2 protein and inhibits viral replication [7]. For the past twenty years, several RNA-targeted therapies have been marketed (Table 1). Nowadays, with the development of next-generation chemistry and delivery techniques, more than 100 clinical trials are currently ongoing, covering a wide range of diseases, including cancer, neurological disorders, metabolic disorders, and infectious diseases (Table 2, Supplementary Table S1). ASOs and siRNA therapies are the most popular strategies. These oligonucleotide-based drugs obtain improved potency through chemical modifications and delivery vectors [8,9]. However, there are still some challenges to overcome, such as off-target toxicity and immunogenicity, before they can be extensively applied to routine clinical practice [10,11,12]. Alternatively, RNA-targeted small molecules have a few advantages over the oligonucleotide therapies, in terms of their flexibility of drug delivery, manufacture as well as administration [13].

In this review, we will explore the discovery process of FDA-approved and other pending therapies for spinal muscular atrophy (SMA) that target survival motor neuron (SMN) pre-mRNA and splicing regulatory proteins. We will also present two examples of potential RNA-targeted therapies, which could work through regulating RBPs and RNA metabolism—Lin28 and let-7; as well as HuR and Musashi proteins, and their RNA targets. We will review the drug discovery processes targeting these RNA-protein interactions in depth, which utilised high-throughput screenings (HTS). The focus will be placed on the screening strategies and the interplays between the RBP, its relevant drug, and the RNA target. Finally, we will highlight several other discoveries towards RNA-targeted therapies of human diseases. Together, this review provides insights into the pathways involved in uncovering novel, RNA-targeted therapies.

## 2. Targeting Lin28/let-7 Pathway

### 2.1. Let-7 Is Post-transcriptionally Regulated by Lin28

MicroRNAs (miRNAs, miRs) are a class of short non-coding RNAs that silence gene expression post-transcriptionally. To date, more than 2000 miRNAs have been identified in *Homo Sapiens* [24]. In animals, miRNA transcript, the hairpin-like primary miRNA (pri-miR), is cleaved by the microprocessor. The microprocessor complex is assembled by the RNase III Drosha, and DiGeorge syndrome critical region 8 (DGCR8) proteins. The resultant stem-loop product is a precursor miRNA (pre-miR), which is then transported to the cytoplasm, where it is further processed by the RNase III Dicer [25]. Dicer cleavage removes the terminal loop of pre-miR and produces a miRNA duplex. Only one strand becomes the mature miRNA, which complexes with Argonaute proteins as part of the miRNA-induced silencing complex (mRISC) [26]. The mRISC triggers translation repression or mRNA degradation through the base-pairing between the miRNA and the target mRNA [27].

**Figure 1:**
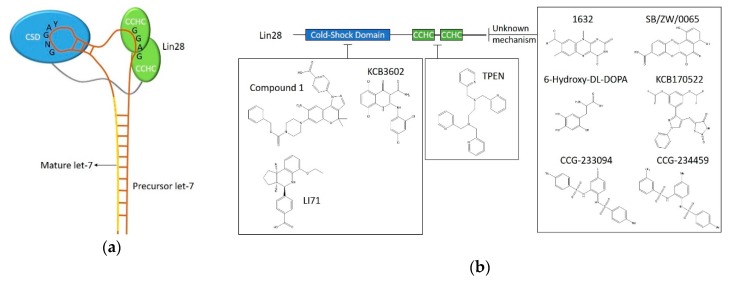
Targeting the Lin28/let-7 pathway. (**a**) Interactions between Lin28 and pre-let-7. Cold-shock domain (CSD) and tandem CCHC motifs of Lin28 are shown in blue and green, respectively. Pre-let-7 is shown in orange and mature let-7 in yellow. CSD and CCHC domains bind to the terminal loop of pre-let-7 at GNGAY and GGAG motifs, respectively. (**b**) Disruptors of Lin28/let-7 identified from HTS. CSD and CCHC motifs of Lin28 are shown in blue and green, respectively. Hit compounds identified through HTS as being disruptors of Lin28/let-7 are displayed. The targeting domains are notified. Disruptors with unclear mechanisms are pointed out.

Let-7 was first discovered as a regulator of stem cell differentiation in *C. elegans*. There are nine mature let-7 miRNAs in the human let-7 family, encoded by 12 different genomic loci [28]. The biogenesis of let-7 is blocked by Lin28 proteins through the interactions with the let-7 terminal loop [29,30,31,32]. Let-7a-3 is an exception that evades Lin28A regulation [33]. Lin28 is abundantly expressed in undifferentiated cells and its expression declines during differentiation, which is reciprocal to the mature let-7 level [34]. Lin28A and Lin28B are the two paralogs in human cells, sharing a conserved cold-shock domain (CSD) and two tandem Cys-Cys-His-Cys (CCHC) zinc finger domains. The CSD domain and CCHC motifs bind the terminal loop of let-7 precursors at the GNGAY and GGAG motifs, respectively [35] (Figure 1a). Lin28A recruits TUT4 (terminal uridylyltransferase 4) together with E3 ligase Trim25 to induce a 3’-uridylation of pre-let-7; therefore, preventing Dicer processing [36,37]. Alternatively, Lin28B retains pri-let-7 in the nucleoli, where the microprocessor is not available [38].

Let-7 inhibits the expression of many oncogenes, such as HMGA2, KRAS, and MYC, marking its function as a general tumour suppressor for carcinomas, exemplified by its role in lung cancer and multiple myeloma [28,39,40,41]. Interestingly, the let-7 family also targets the 3’-UTR of Lin28A and Lin28B, downregulating the expression of Lin28 proteins in embryonic neural stem cells [32]. This suggests a negative feedback mechanism of the Lin28/let-7 pathway. Lin28A/Lin28B overexpression is found in a plethora of advanced carcinomas, where the let-7 level is frequently repressed [42,43,44]. Lin28 mediated let-7 inhibition leads to the let-7 targets being reinstated. This contributes to accelerated tumorigenesis, increased metastasis, as well as resistance to radiation and chemotherapies [45,46]. Based on this evidence, the interruption of Lin28/let-7 is considered an attractive therapeutic approach [39]. Here we will discuss the most recent drug screening techniques targeting this pathway, as well as the performance of the hit compounds (Figure 1b).

### 2.2. FRET-Based HTS Targeting Lin28/let-7

Fluorescence resonance energy transfer (FRET)-based screenings are widely used in the study of protein–protein interactions and have recently been applied in RNA-protein targeted screenings. Normally, the two components are tagged with a donor or acceptor fluorophore, respectively. Quenching of the donor fluorescence or enhancement of the acceptor fluorescence can be detected, allowing interpretation of the interactions. The first small molecule disruptor of Lin28/let-7 was identified through FRET screening of 16,000 drug-like small molecules from the Maybridge Hitfinder library [47]. Instead of using two fluorophores, Lin28B was co-produced with EGFP (enhanced green fluorescent protein), while pre-let-7-2a was conjugated with black-hole-quencher 1 (BHQ-1). The fluorophore-quencher design enhanced the assay’s sensitivity by improving the FRET effects without increasing the spectral bleed-through. Fourteen hits were obtained after two rounds of HTS, among which the compound 1632 (N-Methyl-N-[3-(3-methyl[1,2,4]triazolo[4,3-b]pyridazin-6-yl)phenyl]acetamide) was verified to upregulate the endogenous let-7 by two to three fold, in Huh7 cells. This was done using the Renilla luciferase let-7 reporter assay and quantitative reverse transcription PCR (qRT-PCR) [47]. The compound 1632 also showed an obvious disruptive effect in the Lin28A/pre-let-7a-2 ELISA with an IC50 of 8 μM. It acts by binding to Lin28 proteins but not RNA [47]. The binding domain has not been clarified yet. Notably, this compound induced differentiation-like morphologies in murine ESCs and inhibited tumorigenesis in human cancer cell lines, which are consistent with the morphological changes triggered by upregulated let-7 [47]. An independent research group claimed that 1632 treatment inhibited breast tumour growth and metastasis in mouse models. This was achieved by blocking the Lin28B/MYC/miR-34a axis, which regulates tumour glucose metabolism and the acidity of the microenvironment [48]. However, despite being a key regulator of this pathway, let-7 level upregulation after 1632 treatment was not reported in this article. Thus, more evidence is required to validate that the Lin28/let-7 pathway is the mechanism through which 1632 displays its anti-cancer properties.

Another FRET-based screening assay labelled the terminal loop of pre-let-7a-1 at the 3’-end with the quencher molecule BHQ-2 [49]. The fluorophore Cy3 was attached to the flexible linker between the CSD and CCHC domains of Lin28A through unnatural amino acid mutagenesis. This labelling strategy achieves closer proximity between the FRET donor and acceptor without hampering the binding activity of the protein, thus enabling a higher quench efficiency (85%) and sensitivity (Z’ factor = 0.94). A library containing a variety of 4,500 drug-like molecules was previously constructed using a privileged substructure-based synthesis strategy [50]. A benzopyranylpyrazole-based hit compound (denoted as compound 1) with micromolar IC50s was identified in the FRET screening followed by validation with an electrophoretic mobility shift assay (EMSA) [49]. This hit compound blocks the CSD of Lin28A, as shown in surface plasma resonance (SPR). Apart from working as an antagonist against Lin28A/let-7a-1, the compound was also validated to play a similar role against Lin28B and let-7g in EMSA. The antagonist decreased both Lin28A and Lin28B bound on pre-let-7a in RNA pulldown assays. Moreover, it facilitated mature let-7g production in the Dicer processing assay. A twofold increase was observed in the levels of 6 mature let-7 family members in JAR human choriocarcinoma cells. Additionally, compound 1 showed no significant rescue of let-7 in Lin28 deficient or Lin28 knockdown cells [49]. According to the luciferase reporter assays and western blot analysis, the oncogenic proteins targeted by let-7 (HMGA2, c-Myc and Ras) were downregulated by the Lin28/let-7 disruptor in JAR cells, emphasising its function in facilitating cellular let-7 biogenesis [49]. The Park group carried out another HTS targeting Lin28/let-7 from an 8,400-compound library (Korea Chemical Bank) using the same FRET-based platform [51]. The most potent hit, KCB3566, showed an IC50 of 11 μM, in EMSA. By analysing KCB3566 analogues from the entire KCB libraries (430,000 compounds), KCB3602 was selected as the best candidate by effectively disrupting Lin28A binding to the terminal loop of pre-let-7g and pre-let-7-a-1 in EMSA (IC50 = 4.8 μM and 11 μM, respectively) [51]. SPR showed KCB3602 bound to the CSD domain of Lin28A with a Kd of 5.9 μM. Compared with the hit from their first screening, KCB3602 facilitated the biogenesis of let-7 and inhibited downstream oncogenic proteins at a lower concentration (10 μM) [51]. Moreover, the novel disruptor displayed anti-cancer activities by relieving paclitaxel-resistance and inhibited sphere-like growth in JAR cells [51].

### 2.3. FP-Based HTS Targeting Lin28/let-7

Another common screening strategy is fluorescence polarisation (FP). Excited by the corresponding polarised light, a fluorophore linked to an unbound ligand depolarises the light emitted. While the ligand is associated with a protein, the polarisation is retained due to a slower rotation of the bound ligand. The affinity of ligand binding can be measured according to the difference of the polarised light emitted by the fluorophore [52]. A previous FP-based HTS included 2,768 molecules, most of which came from two commercially available libraries. A small portion of molecules was designed to specifically target nucleic acid structures [53]. In this screen, the terminal loop of pre-let-7g with natural flanking nucleotides (5-nt each at both ends) was labelled with a 3’-fluorescein. The FP was acquired in the presence of the N-terminal glutathione S-transferase (GST) tagged Lin28A. After two repeats of the FP screening, 21 hits gave inhibition consistently greater than 50%. However, only four out of 21 hits showed equivalent inhibitive effects on full-length Lin28A/pre-let-7g in EMSA, and only two compounds restored the mature let-7g level in the in vitro Dicer processing assay when the biogenesis of pre-let-7g is partially inhibited by Lin28A [53]. The two hit compounds are 6-hydroxy-DL-DOPA, a dopamine precursor and SB/ZW/0065, a benzo[a]phenoxazine. Whether these two hits could function at a cellular level remains unvalidated. Furthermore, whether they are applicable for use in other let-7 family members is unknown.

A more recent FP-based screening was conducted using an extensive 101,017-compound pool, combining six commercially available libraries [54]. The FP system was composed of the terminal loop of pre-let-7f-1 with a 3’-FAM, and a truncated human Lin28A (with functional CSD and CCHC domains) bearing a single mutation which enhances the let-7 binding affinity to the tandem CCHC pocket. The modifications allowed a high sensitivity with a Z’ factor = 0.83. Twenty-seven hits were identified with reproducible IC50s between 200 nM and 10 μM in the FP-based HTS. Six of the hits could inhibit mouse Lin28A/TUT4 mediated pre-let-7g uridylation. Interestingly, the candidate compounds have different working mechanisms. TPEN (N,N,N′,N′-tetrakis(2-pyridinylmethyl)-1,2-ethanediamine) is a zinc chelator that interrupted the pre-let-7 binding at the CCHC zinc knuckle motifs. Another potent inhibitor LI71 interacted with the CSD pocket of Lin28A and upregulated a range of mature let-7 levels in embryonic and leukaemia cells, as detected by dual luciferase reporter assay and qRT-PCR. The let-7 levels detected in embryonic cells by qRT-PCR reached an impressive three to six-fold upregulation, despite massive variations between the technical repeats [54].

### 2.4. Other HTS Targeting Lin28/let-7 and Conclusions

A novel fluorescence intensity-based binding assay (FL assay) was developed recently to screen modulators of RNA-protein interaction, as protein-binding induced fluorescence enhancement can be detected when nucleic acid fragments are conjugated with environment-sensitive organic fluorophores [55]. Based on this FL assay, the interaction between Lin28A and 3’-TAMRA-labelled terminal loops of pre-let-7-g was investigated using representative compounds from KCB, natural products and drug-like small-molecule collections (Z’ prime = 0.56). The reliability of the method was confirmed by three previously identified Lin28/let-7 disruptors, including the aforementioned hit KCB3602 discovered by the same group [55]. The screening generated four initial hits, including KCB170522, luteolin, rhynchophylline, and tenuifolin. However, only KCB170522 increased cellular let-7 levels in JAR cells and inhibited the expression of let-7 targeted proteins (HMGA2, c-Myc and Ras) [55]. Compared with FRET and FP, the FL assay-based screening is simple, with comparable efficiency, at least in this case. Larger scale screening is required to prove the feasibility and effectiveness of this novel screening approach.

Click chemistry is often applied in HTS by joining two biomolecules efficiently. The Garner group developed a catalytic enzyme-linked click chemistry assay (cat-ELCCA) to monitor protein–protein interaction and miRNA biogenesis in a high-throughput format [56,57,58]. Based on cat-ELCCA, an HTS was carried out targeting Lin28/let-7 interaction by screening 127,007 small molecules from five libraries (LOPAC, Prestwick, Maybridge, ChemDiv, and the University of Michigan Chemistry). Murine Lin28A was immobilised and incubated with pre-let-7d carrying a 5′-trans-cyclooctene (TCO) click chemistry handle. This was followed by click chemistry with methyltetrazine-conjugated horseradish peroxidase (mTet-HRP). Lin28/let-7 interaction was detected by measuring chemiluminescence signals in the presence of an HRP substrate [59]. The screening exhibited a Z’ prime of 0.5 and generated 1,468 initial hits with more than 25% inhibition. Only two N,N′-(1,2-phenylene)-dibenzenesulfonamide derivatives, namely CCG-233094 and CCG-234459, showed dose-dependent inhibition in both cat-ELCCA and EMSA with micromolar IC50s. The binding between Lin28A and these two compounds was confirmed by SPR [59]. Nevertheless, whether the molecules can upregulate let-7 level and lead to any biological consequences in the cellular context remains to be tested. This click chemistry-based HTS approach provides fluorescence-free alternatives for screening protein-RNA regulators.

Lin28/let-7 is the best characterised RBP-miRNA interaction. These HTS are important examples for drug discovery of RBP-miRNA modulators. Our group have identified Lin28A as a negative regulator of the biogenesis of miR-9, through a distinct mechanism [60]. Additionally, hnRNP A1 negatively regulates let-7a levels [61,62,63]. We also found that miR-7 was modulated by cooperative effects between two RBPs, which will be described in detail later [64]. This means that all identified Lin28/let-7 compounds will have to be tested for their specificity and efficiency in cellular and animal models before going forward in anti-cancer therapy trials.

## 3. Spinal Muscular Atrophy (SMA) RNA-Targeted Therapy

### 3.1. Pre-mRNA Splicing and SMA

**Figure 2:**
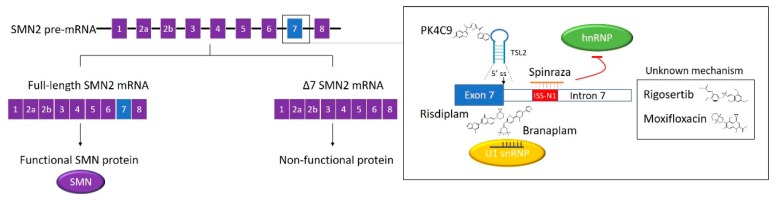
RNA-targeted therapy of SMA. The exons of SMN2 pre-mRNA are shown as squares and introns as lines. Alternative splicing with exon 7 inclusion generates full-length SMN2 mRNA and functional protein, while the loss of exon 7 results in non-functional protein. The junction between exon 7 and intron 7 is enlarged. SMN2 splicing modifiers and the working mechanisms are indicated. Spinraza is an approved ASO drug binding at ISS-N1, preventing the hnRNP interaction. Risdiplam and branaplam stabilise the U1 snRNP binding to the 5’ss of exon 7. PK4C9 acts at TSL2, a secondary structure at the exon 7/intron 7. Rigosertib and moxifloxacin correct SMN2 splicing with unknown mechanisms.

Pre-mRNA splicing is a critical process in the production of functional mature mRNA in eukaryotic cells. During the process, introns are removed, and exons are joined. Pre-mRNA splicing is catalysed by a macromolecular ribonucleoprotein complex known as the spliceosome. The major spliceosome is composed of five small nuclear RNAs (U1, U2, U4, U5, and U6), which combine with proteins to form small nuclear ribonucleoproteins (snRNP). These snRNPs recognise conserved sequence elements at 5’ splice sites (5’ss), 3’ss and branch points [65]. Alternative splicing occurs in 95% of human genes, increasing protein diversity from the limited number of genes [66]. Trans-acting RBPs such as serine-arginine-rich proteins (SR proteins) and heterogeneous nuclear ribonucleoproteins (hnRNPs) modulate alternative splicing by interacting with cis-elements including the exonic splicing enhancer (ESE), intronic splicing enhancer (ISE), exonic splicing silencer (ESS), and intronic splicing silencer (ISS). In most cases, SR proteins work as activators, while hnRNPs act as suppressors. However, these RBPs can play either role in a context-dependent manner [67]. Aberrant splicing is often implicated in severe diseases such as cancer, Duchenne muscular dystrophy (DMD) and spinal muscular atrophy (SMA). Therefore, correction of alternative splicing has the potential to be developed into various new therapies [68,69]. Here we will review the development of SMA therapies targeting alternative splicing, with a particular focus on the discovery of the first SMA drug (Figure 2).

SMA is an autosomal recessive disorder characterised by motor neuron degeneration, leading to progressive muscle weakness, atrophy and paralysis. SMA occurs in 1 in 10,000 new-borns and is dominated by SMA type I (Werdnig–Hoffmann disease), a fatal, infantile-onset subtype [70,71]. The majority of SMA is caused by the loss of survival motor neuron (SMN) proteins, as a result of homozygous deletion or point mutation in the SMN1 gene. SMN2 also encodes SMN protein, but only produces a small proportion of functional full-length proteins. Compared to SMN1, SMN2 bears a single, silent C to T transition in exon 7, which induces exon 7 skipping during alternative splicing. Therefore, the resultant truncated SMN protein is inactive and degraded rapidly [72]. The SMN2 copy number is generally inversely correlated to clinical severity [73,74].

### 3.2. The Discovery of the First SMA Drug

Spinraza (nusinersen) is the first FDA approved drug for the treatment of SMA. The drug is a 2′-O-(2-methoxyethyl) (MOE) phosphorothioate-modified ASO. The modification endows the ASO drug with an improved in vivo stability [75]. The discovery of Spinraza started with a two-step ASO walk strategy by the Krainer group aiming to find modulators of SMN2 exon 7 alternative splicing [76]. Nine ASOs were designed in a 5-nt step along the entire exon 7 in the initial coarse screen. Two inhibitory regions of exon 7 inclusion were identified using in vitro (RNA-based) and in vivo (cell-based) splicing assays. The following screen-tested 39 ASOs, ranging from 12-nt to 18-nt, complementary to the two putative targeting pre-mRNA regions. Two of the most potent exon 7 inclusion stimulatory ASOs promoted the expression of corresponding reporter proteins in HEK293 cells, as well as the full-length SMN protein in primary fibroblasts derived from a type I SMA patient [76]. Following this, in collaboration with Ionis Pharmaceutics, the Krainer group performed a second ASO screening, targeting intronic sequences flanking exon 7 [77]. Twenty 15-nt ASOs were tested in the initial screen, with a 10-nt overlap between the neighbouring ASOs, covering 60-nt at the end of intron 6 and the start of intron 7, respectively. The ASOs 11–25, targeting intron 7, showed the strongest exon 7 inclusion effects in both in vitro and in vivo splicing assays [77]. The target region was characterised as an intron 7 ISS, which showed a large overlap with a previously identified silencer termed ISS-N1(10^th^–24^th^ position in intron 7) [78]. Thirty-eight ASOs of different lengths targeting the ISS were tested with the cell-based splicing assay. The most potent 18-mer and 15-mer were ASO 10–27 and ASO 9–23, respectively. The two ASOs were investigated in SMA type I patient fibroblasts. They presented more efficient stimulatory effects in exon 7 inclusion and SMN protein production when compared with the two best ASOs targeting exon 7 from the first screen [77]. After extensive screening and optimisation, the MOE-modified ASO 10–27 was selected as the lead, as it showed the greatest therapeutic potential [79]. The lead ASO was subsequently tested in different SMA mouse models. It achieved promising therapeutic effects by correcting SMN2 splicing, rescuing necrosis, and improving motor function and survival [77,79,80,81]. The optimised lead ASO was approved by the FDA in 2016 and is now sold under the name of Spinraza. The drug is administrated to the human central nervous system through intrathecal injection.

Spinraza has been shown to improve various clinically important outcomes, including increased survival and enhancement of motor function. This is true for both early-onset type I disease and later-onset type II and III diseases [82,83]. As the drug is still recently approved for clinical application, the permanency of the effects of the drug is currently uncertain. However, it is hoped that as further data becomes available, this picture will be increasingly clear. Furthermore, when accounting for the considerable benefits already proven through the relatively short-term application of the therapy, the potential for effective long-term therapy is promising. This is supported by the data provided by the NUTURE study. Children in this study, treated with Spinraza while pre-symptomatic, showed significant increases in life-expectancy and motor function when compared with what would be expected for untreated children. Moreover, the study marks the potential benefit of initiating treatment before the onset of symptoms [84].

It is clear that Spinraza functions by sequestering ISS-N1, but the mechanism of how ISS-N1 works as a negative SMN2 splicing regulator remains contentious. It was previously assumed that the two putative hnRNP A1/A2 binding motifs in ISS-N1 were responsible for the inhibitory effects [77]. However, recent evidence showed hnRNP A1 interacting with both binding sites through two RNA recognition motifs (RRMs). Both interactions were essential to mediate the splicing repression [85]. Moreover, an increasing number of studies have suggested other motifs in ISS-N1, and secondary RNA structures beyond the ISS-N1 region, are involved [75,86]. ASOs targeting other cis-acting elements, as well as splice sites of neighbouring exons were explored [87,88]. A greater understanding of these should facilitate complete comprehension of the mechanism involved in SMN2 splicing correction and serve as potential therapeutic targets for different types of SMA.

Recently, a gene replacement therapy named Zolgensma (onasemnogene abeparvovec-xioi, AVXS-101) was approved by the FDA for paediatric SMA patients with bi-allelic mutations in the SMN1 gene [89]. Zolgensma delivers human SMN1 gene to motor neurons by adeno-associated virus (AAV) intravenously, facilitating the production of functional SMN proteins. Despite there having been no study directly comparing Zolgensma to Spinraza to date, several papers have provided indirect comparisons. In general, these report a no difference in clinical efficacy, or a slight benefit leaning towards the use of Zolgensma [90]. Similarly, in terms of cost-effectiveness, the two therapies would appear to be similar, with Zolgensma again potentially being slightly cheaper long-term [91]. On the whole, both therapies would appear to be economically beneficial when compared to no therapy, on top of the substantial benefits to SMA patients [92].

### 3.3. Small Molecule SMA Drug Candidates Correcting SMN2 Splicing

Currently, two orally bioavailable small molecules targeting SMN2 splicing correction, risdiplam and branaplam, are under evaluation in clinical trials. If approved, these drugs would advance SMA treatment and improve patient accessibility beyond Spinraza and Zolgensma.

Risdiplam (RG7916) is structurally optimised from RG7800, the first small-molecule SMN2 splicing modifier to enter human clinical trials [93]. Unfortunately, the trials were suspended due to ocular toxicity in long-term animal studies [94,95]. The predecessor compound of RG7800 was identified from a phenotypic HTS. The screening was based on an SMN2 minigene luciferase assay in HEK293H cells using a proprietary library (PTC Therapeutics) containing more than 200,000 small molecules [96]. Only the full-length SMN2 minigene mRNA was in frame with the firefly luciferase sequence, so exon 7 inclusion enhancers would generate increased luminescence. The positive hits were validated using qRT-PCR and semi-quantitative end-point RT-PCR to confirm the increase of full-length SMN2 mRNA production. The identified small-molecule splicing modulators (SMN-C class compounds) could increase full-length SMN2 protein levels in fibroblasts from different types of SMA patients. This was highly selective towards SMN2 transcript, as assessed by RNA sequencing [96]. These small molecules showed good oral bioavailability, as well as improved motor function and longevity in SMA mouse models [96,97,98]. NMR structures indicated that SMN-C5 specifically stabilised a bulged adenine at the exon-intron junction in the helix formed by U1 snRNA and exon 7 5’ss; therefore, promoting U1 snRNP recognition and converting the weak 5’ss into a stronger one [99]. This introduces a new concept for gene-specific alternative splicing correction termed as 5ʹ splice site bulge repair. Moreover, SMN-C5 worked cooperatively with the splicing regulatory network, thus, displaying higher potency in cellular models than *in vitro* assays [99]. Additionally, previous evidence indicated that SMN-C class compounds bound to the ESE2 region on exon 7, through potential mechanisms relevant to SMN2 splicing activators, including hnRNP G, far upstream element-binding protein 1 (FUBP1), and KH-type splicing regulatory protein (KHSRP) [100,101]. However, ESE2 binding is not the dominant cause of splicing correction, and the action mode of these RBPs are not fully understood [99]. Compared to RG7800, risdiplam showed enhanced potency and improved safety in preclinical studies [93]. The drug candidate was well tolerated in a Phase 1 clinical study and is being investigated in Phase 2/3 studies for all types of SMA [102]. Risdiplam is now under priority review for possible approval by the FDA.

Branaplam (LMI070) was discovered from a 1.4 million-compound library (Novartis) using a pair of opposite SMN2 minigene luciferase reporters expressed in an NSC34 motor neuron cell line [103]. The screen resulted in less than 1.0% positive hits. These hits showed significant elevation of full-length SMN2 reporter luciferase signal and a concomitant reduction of Δexon 7 reporter signal. The average Z’ factor and the robust Z’ factor were 0.59 and 0.71, respectively. The SMN2 splicing correction activity of the hits was confirmed using qRT-PCR. Increases in SMN protein levels were evaluated in SMNΔ7 mouse myoblasts [103]. Based on a pyridazine core functional group identified from the hit compounds, branaplam was developed through medicinal chemistry optimisation. This provided advantages in potency, bioavailability, and safety [104]. This potential drug enhances full-length SMN protein production and extends survival in a severe SMA mouse model [103]. Branaplam shares a similar pharmacophore with the SMN-C compounds [100]. It also acts by stabilising the dsRNA interaction between U1-snRNP and the 5’ss of SMN2 exon 7. This may occur in the major groove proximal to the nGA motif at the exon portion of the predicted 5′ss junctions [103]. The precise action site of branaplam at 5’ss is slightly different from SMN-C5 [100]. Branaplam is currently in a Phase 1/2 clinical trial for type I SMA treatment.

### 3.4. Other SMN2 Splicing Modifiers Identified by HTS and Conclusions

Secondary RNA structures are potential targets for small molecule splicing modifiers. Terminal stem-loop 2 (TSL2) is a 19-nt RNA hairpin formed at the exon 7/intron 7 junction of SMN2, with its 3’ end partially overlapping with 5’ss of exon 7 [105]. Prior to an HTS targeting TSL2, it was confirmed that conformational changes in TSL2 could enhance SMN2 exon 7 inclusion [106]. From a 3,000-compound in-house library, 304 small molecules were selected for the screening using in silico filtering, as the molecules bear RNA recognition scaffolds, including indole, 2-phenyl indole, 2-phenyl benzimidazole, and alkyl pyridinium [106]. The HTS was carried out using a fluorescence displacement (FD) assay, with the help of a fluorescent indicator TO-PRO [106]. The fluorescence intensity of unbound TO-PRO is negligible, but it will increase by 500-fold when TO-PRO binds to a structured RNA, which is the TSL2 hairpin in this case. The replacement of the dye with an RNA ligand will result in a loss of fluorescence [107]. The screening generated 54 initial hits with a Z’ factor of 0.7. The hit-induced TSL2 conformational change was assessed by a 2AP assay, where TSL2 was labelled by a fluorescent structural probe 2-aminopurine [106]. Nineteen candidates entered a secondary screen using a SMN2 minigene reporter assay in HeLa cells. Among these, homocarbonyltopsentin (PK4C9), a marine natural molecule showed the strongest exon 7 inclusion effect with 25 μM EC50 [106]. PK4C9-induced splicing correction was also effective in SMA fibroblasts and Drosophila motor neurons. Importantly, the molecule raised functional SMN protein level and reversed SMN deficiency phenotypes in SMA cells [106]. NMR and molecular modelling studies showed PK4C9 directly induced an opening conformation of TSL2, leading to improved accessibility of 5’ss to splicing factors. Furthermore, PK4C9 indirectly altered a pentaloop conformation of TSL2 into a triloop. The triloop conformation is potentially favoured by exon 7 splicing [106]. With the realisation that secondary RNA structures, critical for SMN splicing, are druggable, new avenues may open up for SMA therapy.

Luciferase SMN2 minigene reporters were extensively applied in the previous HTS of SMN2 splicing modulators [94,108]. Rigosertib, a well-known PLK (Polo-like kinase) inhibitor, was identified as the best candidate from a drug screening using the SMN2 reporter cell lines [109]. Rigosertib induced exon 7 inclusion and SMN protein restoration in motor neuron progenitors differentiated from SMA patient-derived induced pluripotent stem cells (SMA iPSCs-pMN). SMA-related pathological phenotypes were rescued by the candidate compound in this human SMA model cell line [109]. Another very recent HTS described moxifloxacin as an exon 7 inclusion enhancer using a SMN2 minigene reporter in Drosophila motor neurons. The effects were validated in patient-derived fibroblasts [110].

After the development and approval of Spinraza, the field of RNA-targeted drug development for the therapy of SMA has expanded exponentially. The development of ASO drugs raised hope for RNA-targeted therapies aimed at RBPs, previously considered undruggable. Additionally, the identified splicing modifiers are useful tools to study the mechanism of how RBPs regulate alternative splicing. A deeper understanding of the mechanism will, in turn, facilitate the development of more specific therapies. Finally, minigene reporters are good models for screening splicing modulators, if the hits are validated using more physiologically relevant models.

## 4. Regulation of HuR and Musashi Proteins Binding to RNA

### 4.1. miR-7 Biogenesis is Controlled by HuR/MSI2 Complex

MicroRNA-7 (miR-7) is a brain-enriched miRNA. Dysregulation of miR-7 could contribute to severe diseases, especially neurological disorders [111]. In glioblastoma (GBM) and Parkinson’s disease (PD), the miR-7 level is often downregulated [112,113]. Restoration of miR-7 levels could have therapeutic potential, as miR-7 targets the mRNA of epidermal growth factor receptor (EGFR) and α-synuclein, which are overexpressed in brain tissue from GBM and PD patients, respectively [112,113,114]. Moreover, evidence has shown that PD is a risk factor in GBM [115,116].

**Figure 3:**
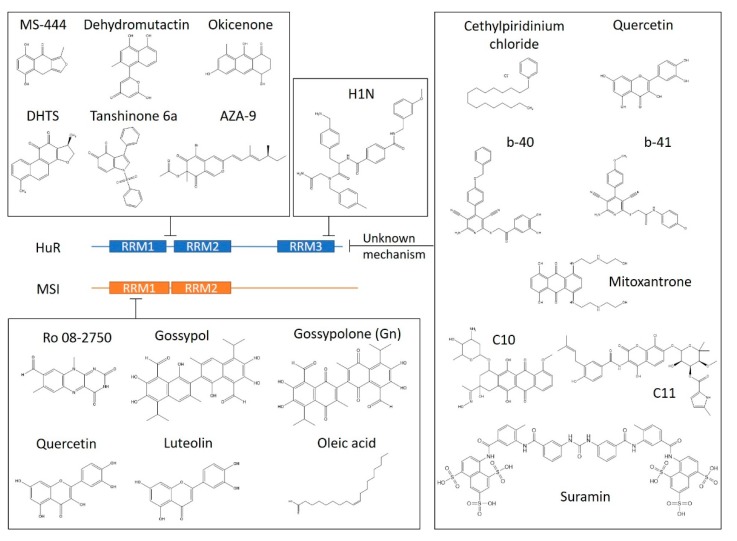
Regulation of HuR and Musashi proteins (MSI) binding to RNA. RNA recognition motifs (RRMs) of HuR are shown in blue, and RRMs of MSI are shown in orange. Small molecules interrupting RNA binding activities of HuR or MSI are displayed. The known targeting RRMs are indicated. HuR inhibitors with unknown mechanisms are shown.

Our group has identified that an RBP HuR (ELAVL1) binds to the terminal loop of pri-miR-7 and recruits another RBP MSI2 (Musashi RNA-binding protein 2) to inhibit the biogenesis of functional miR-7 [64]. We also showed that the pri-miR-7/HuR/MSI2 complex could be disrupted by oleic acid (OA), a monounsaturated fatty acid (MUFA), resulting in elevated miR-7 levels [117]. Thus, disruptors of pri-miR-7/HuR/MSI2 could become promising drug candidates for GBM and PD. However, there are no relevant HTS reported so far, and the tagetome of miR-7 remain unknown. Here, we will review the inhibitors of HuR and Musashi proteins binding to mRNAs, as well as the corresponding HTS strategies, which might provide avenues for finding miR-7 enhancers by interrupting the pri-miR-7/HuR/MSI2 complex (Figure 3).

### 4.2. HuR Binds AU-Rich Elements

HuR is expressed ubiquitously across human tissues. The protein often stabilises mRNAs bearing AU-rich elements (AREs) in their 3’-UTRs. Most of the targeted ARE-RNAs encode oncogenic proteins, highlighting a crucial role of HuR in regulating the development and progression of multiple human cancers [118]. HuR inhibition can sensitise tumour cells to cancer therapies [119,120]. Interestingly, HuR represses SMN2 splicing by binding to the -44 region in intron 6, implying its potential to become an SMA target [121]. HuR contains three RRMs. The N-terminal tandem RRMs (RRM1 and RRM2) are responsible for ARE binding, and the C-terminal RRM (RRM3) can bind ARE and poly-A, with suggested roles in protein–protein interaction, dimerisation, post-transcriptional modifications, and stabilisation [122,123]. However, the roles of RRM3 in the context of full-length HuR remain controversial [122,123,124].

### 4.3. HTS Targeting Different RRMs of HuR

A confocal fluctuation spectroscopic screening assay was performed with recombinant HuR_12_ (containing only RRM1 and RRM2), and tetramethylrhodamine (TMR) labelled ARE-RNA (unspecified) using a 50,000-compound library composed of microbial, mycological, and plant extracts (Novartis) [125]. In complex with HuR, the fluorescently labelled ARE-RNA should have a slower rotation, resulting in an increased fluorescence anisotropy measured by 2D-FIDA (two-dimensional fluorescence intensity distribution analysis). The Z’ prime of this HTS was 0.86. Fifty primary hits were identified with more than 40% inhibition of HuR-induced anisotropy increase. The active fractions were isolated using reverse-phase HPLC, and five compounds were selected from the fractions as they showed the strongest HuR-RNA inhibition according to NMR, IR, and MS [125]. Three out of the five molecules inhibited full-length HuR from binding to the AREs of interleukin-2 (IL-2), tumour necrosis factor-α (TNF-α), cyclooxygenase 2 (Cox-2), and IL-1β in a concentration-dependent manner, as monitored by 2D-FIDA [125]. The three nanomolar inhibitors were dehydromutactin, MS-444, and okicenone. They interfered with HuR homodimerisation and cytoplasmic trafficking; prevented HuR-mediated ARE-mRNA stabilisation in the cytoplasm, and decreased ARE cytokine expression in primary human monocytes [125]. MS-444 is the most potent compound, and it has been proven to effectively disrupt HuR targeting to different mRNAs by several independent research groups [120,126,127,128]. Notably, the anti-cancer effects of MS-444 through HuR inhibition were observed in malignant pancreatic [120,129], colorectal [130,131], melanoma [132], and glioma cells [133] in vitro or in xenograft mouse models. The drug development of MS-444 by Novartis has not been reported yet, so the future of this HuR inhibitor is uncertain.

Confocal nanoscanning (CONA) screening from one-bead-one-compound libraries was used to look for HuR ligands, specifically targeting RRM3 and elucidate the controversial functions of the orphan RRM3 pocket [122]. Eighty-nine thousand heterocyclic compounds were immobilised on 90 μm TentaGel beads. Each compound class comprised four different building blocks, representing a total of 2.2 million compounds. HuR binding hits were detected as fluorescent rings when Cy5 tagged HuR was captured by compounds on the beads. The ring intensities were proportional to the amount of bound HuR. HuR RRM3 selective hits (binding full-length HuR but not HuR_12_) with the highest intensities were found in sub-libraries of pyrrole and dicarboxylic acid scaffolds [122]. Forty-six molecules were identified from the isolated hit beads by MS decoding, four of which were found repetitively. The four hit compounds were resynthesised, and their selective binding to HuR RRM3 was confirmed in CONA. Compound H1N belongs to the dicarboxylic acid scaffold sub-library and showed the highest binding affinity in the CONA screen, as well as competition assays [122]. With the help of H1N bead resin in CONA, an ATP-binding pocket associated with 3′-terminal adenosyl transferase activity was identified in HuR RRM3 [122]. Notably, the ATP-binding affinity is not detected in other conventional binding assays using isolated RRM3 [123]. The on-bead screen strategy allows the identification of low affinity but specific ligands. CONA is also very helpful with the characterisation of binding domains, especially in this case, as both full-length and RRM3 HuR have poor solubilities.

### 4.4. Other HuR/ARE-RNA Disruptors and Therapeutic Effects

A small-scale screen targeting HuR and TNF-α ARE-RNA was completed using EMSA. One hundred seventy-nine compounds, obtained from the Korea Research Institute of Chemical Technology, were tested [134]. Three compounds, quercetin, b-40, and b-41, showed strong disruptive effects with IC50s of 1.4, 0.38, and 6.21 μM, respectively. Quercetin and b-40 could destabilise TNF-α mRNA and decrease levels of secreted TNF-α [134]. Independent research indicated that quercetin displayed significant HuR-mediated IL-6 suppression in vitro and in vivo [135]. EMSA is widely applied to validate protein–RNA interactions. However, due to the technical limitations, EMSA is not efficient for robust, high-throughput screenings.

An AlphaScreen (PerkinElmer)-based HTS was developed to identify inhibitors of HuR binding to TNF-α mRNA [136]. The interaction between biotinylated TNF-α ARE, and c-Myc-tagged HuR was monitored using Streptavidin-Donor beads and anti-c-Myc-Acceptor beads. The Z’ prime was 0.84, and a pilot screen was performed with a 2,000-molecule library (Spectrum Collection, MicroSource Discovery, USA). The library contained clinically approved drugs, natural products and other bioactive components. Ten per cent of hits were rescreened and two compounds, cethylpiridinium chloride and mitoxantrone, were shown to prevent the formation of HuR/TNFα ARE complex by EMSA [136]. HuR binds to ARE-RNA of sex-determining region y-box2 (SOX2) and induces RNA degradation. Mitoxantrone disrupted the interaction between HuR and SOX2 mRNA, resulting in an upregulated level of SOX2 mRNA and protein in mesenchymal stem cells [137]. The same AlphaScreen assay was applied to a small-scale screen from 107 commercially available anti-inflammatory compounds [138]. The most potent inhibitor was DHTS (15,16-dihydrotanshinone-I), which prevented the associations between HuR and TNFα mRNA with nanomolar IC50s. Furthermore, DHTS downregulated both mRNA and protein levels of TNFα and exhibited anti-tumour effects in human breast cancer cells [138]. Structural analysis has shown that DHTS competes with RNA by interfering with both RRM1 and RRM2 of HuR, but the inhibitor is limited to mRNAs with low HuR affinity [139]. The compound inhibited colon cancer cell growth in vitro and in a xenograft mouse model in a HuR-dependent manner [139]. DHTS showed effects similar to MS-444 in glioma, by inhibiting HuR multimerisation and decreasing cellular HuR protein level. Importantly, DHTS showed dose-dependent cytotoxicity towards glioma cells [140]. Based on the structure of DHTS, a series of tanshinone mimics were synthesised. Among these, tanshinone mimic 6a and 6n were more potent HuR/ARE-RNA disruptors when compared to DHTS [141]. 6a and DHTS bind at the same region of HuR, illustrating potential structure-activity relationships (SARs) for HuR inhibitors [141]. The tanshinone mimics affected cell viability in breast and pancreatic cancer cells. However, each of these mimics was less efficient when compared to DHTS [141].

An FP-based HTS has been reported to screen a library of around 6,000 compounds (an in-house library and a library of FDA approved drugs) for HuR/MSI1 ARE-RNA disruptors [142]. The screen generated an initial hit rate of 0.7% with a Z’ factor of 0.79. Six hits (CMLD 1-6) shared a coumarin-derived core and the HuR/ARE disruptive effects were validated using AlphaLISA (Amplified Luminescent Proximity Homogeneous Assay), SPR, the ribonucleoprotein immunoprecipitation (RNP IP) assay, and the luciferase reporter assay [142]. CMLD-2 was the most potent candidate compound among the six hits, with a Ki of 0.35 ± 0.3 μM [142]. Most of the hits displayed moderate cytotoxicity in colon cancer and pancreatic cancer cells. In colon cancer cells, CMLD-1 and CMLD-2 disrupted HuR binding to MSI1 and XIAP mRNAs, and therefore, blocking the Wnt signalling pathway. The anticancer properties were related to apoptosis and autophagy triggered by destabilisation of HuR targets Bcl-2 and XIAP [142]. However, the evidence is not sufficient to prove the SAR of coumarin derivatives as HuR/ARE disruptors and the binding interface has not been confirmed. In particular, CMLD-2 showed therapeutic potentials in lung, breast, and thyroid cancer cells in vitro, with implicated mechanisms relevant to HuR-RNA disruption [143,144,145].

The above FP assay was applied to screen about 2,000 compounds from the National Cancer Institute library (NCI Diversity Set II, Natural Products Set, and Approved Oncology Drugs Set) and an in-house library [146]. The hit compounds were azaphilones, a class of compounds derived from a fungal natural product, asperbenzaldehyde. The most potent candidate was AZA-9 (IC50 = 1.1 μM), which was also effective in disrupting HuR/c-Fos ARE (IC50 = 1.2 μM). Through structural studies, AZA-9 was assumed to competitively bind HuR RRM1 and RRM2 in the RNA binding cleft [146].

Another FP-based HTS was carried out from the NCI library (diversity set V, 1597 compounds) to identify small HuR inhibitors [147]. The screening focused on the interaction between 5’-Fam-labelled c-Fos ARE-RNA and full-length HuR, generating a Z’ factor of 0.8. The primary hits were tested in an NMR-based assay with saturation transfer difference detection (STD-NMR), which provided information on the direct interaction between HuR and the compounds [147].

Using differential scanning fluorimetry (DSF), suramin was recognised as an HuR ligand from an FDA-approved drug library (Pharmakon, 1600 drugs) [148]. Although suramin regulated ARE-mRNAs and the corresponding protein levels in tongue carcinoma cell lines, there is no evidence revealing that the drug functions as a direct HuR/ARE disruptor [148].

### 4.5. Musashi Proteins Recognise Specific RNA Motifs

Human Musashi proteins have two paralogues MSI1 and MSI2, sharing approximately 80% amino acid similarity. Both proteins have two highly conserved N-terminal RRMs, with MSI1 mainly binding (G/A)U_1–3_AGU sequences, whereas MSI2 prefers ACCUUUUUAGAA and UAG motifs [149,150,151]. The C-terminal region of Musashi proteins harbours protein interaction sites, which regulate the translation of Musashi targets [152]. Musashi proteins normally express in stem and progenitor cells and regulate cell differentiation and organ development [153]. Aberrant high expression of Musashi proteins is associated with aggressive tumours, consistent with their roles as translational modulators of some well-recognised oncogenic signalling pathways, including Numb/Notch and PTEN/mTOR [153]. Overexpressed Musashi proteins are found in gliomas, colorectal adenocarcinomas, pancreatic adenocarcinomas, breast cancer, lung cancer, and hematopoietic malignancies [150,153,154,155]. Interestingly, the 3’-UTR of MSI1, but not MSI2, bears ARE targeted by HuR, and MSI1 expression is positively regulated by HuR in GBM [156].

### 4.6. HTS Targeting MSI/RNA Interactions

A 1536-well format FP-based HTS has been developed to identify inhibitors of MSI RNA binding activities [157]. The screen library combines 6,208 compounds from several commercial sources including MicroSource, Prestwick and Tocris. These contain known drugs, experimental bio-actives and natural products [157]. An 8nt-RNA oligo (GUAGUAGU) was selected in this screen, as it was determined to be the shortest oligo with high MSI binding affinity and specificity according to an SYBR-based EMSA assay. The screening was performed using Cy3 labelled RNA oligo and GST fused MSI1 (Z’ = 0.58) or MSI2 (Z’ = 0.61) in a 1536-well plate. Seventeen hits were identified from the initial screen with more than 50% inhibition, five of which inhibited both MSI1 and MSI2. The hits were tested in a secondary screen using a dose-dependent FP assay in a 384-well format. Three inhibitors showed less than 10 μM IC50s towards both Musashi proteins, with slightly higher affinity to MSI1 [157]. The three hits were further validated in an SYBR-based EMSA assay. Following this, one of the hits, Ro 08-2750 (Ro), showed an IC50 of 2.7 ± 0.4 μM to MSI2 in an FP assay. Ro bound directly to MSI2 (Kd = 12.3 ± 0.5 μM) in a MicroScale Thermophoresis (MST) assay [158]. The selectivity of Ro was tested against RBPs sharing conserved RRMs or similar mRNA targets with MSI2, by MST. The molecule showed weak binding (Kd ≈ 200 uM) towards SRSF2 and SYNCRIP, with nearly no binding to HuR. Strong structural and biochemical evidence suggests that Ro interacts with key residues at RRM1 of MSI2 [158]. Importantly, Ro significantly reduces the binding of MSI2-targeted mRNAs, including TGFBR1, c-MYC, SMAD3, and CDKN1A, in an RNA-IP assay. It also inhibits the regulatory effects of MSI2 on the relevant proteins (TGFβR1, c-MYC, SMAD3, HOXA9, and P21) in human leukaemia cell lines [158]. Furthermore, Ro inhibits leukemogenesis in a murine myeloid leukaemia model in vivo, with a concomitant decrease of intracellular c-MYC levels [158]. The efficacy of Ro as an MSI2 inhibitor firmly supports the reliability of this HTS platform, where an artificial RNA oligo, designed by combining MSI binding motifs, is applied as the RNA probe. The optimised RNA probe allows higher selectivity to MSI binding when compared with other natural target mRNAs. This approach may also benefit the screen outcomes because the hits could have greater potential to interrupt universal mRNA targets of a selected RBP when it is desired to achieve a maximum therapeutic effect.

Another FP-based HTS uses a previously identified RNA probe (AGCGUUAGUUAUUUAGUUCG) containing two MSI1 consensus recognition motifs [149]. The interactions between the fluorescein labelled RNA probe and His-tagged recombinant MSI1 dual RRMs (amino acids 7–192) were monitored using FP, during the treatment of two libraries—LOPAC (1280 compounds) and the Chembridge library (30,000 compounds) [159]. The Z’ prime was 0.7 ± 0.2, and four inhibitors were identified from the screen, including OA, an 18-carbon ω-9 MUFA with a Ki of 1.2 ± 0.4 µM. FP and EMSA assays indicated that the RNA binding activity of both MSI1 and MSI2 could be specifically inhibited by ω-9 cis unsaturated fatty acids between 18 and 22 carbons [159]. Computational docking and molecular dynamic simulations elucidated an allosteric inhibition mechanism, where OA induced a conformational change on MSI1 RRM1 and disrupted RNA recognition by key residues [159]. OA exclusively inhibited oligodendrocyte progenitor cell proliferation in the presence of MSI1. Stearoyl-CoA desaturase (SCD), an enzyme which catalyses MUFA synthesis is regulated by MSI1, suggesting a feedback loop of MUFA inhibiting MSI1 [159]. Based on this paper, our group proved that OA facilitated the biogenesis of pri-miR-7 in vitro and in HeLa cells, by remodelling the pri-miR-7/HuR/MSI2 complex. However, this only occurred at relatively high concentrations (500 µM to 1 mM) [117]. Considering OA showed poor bioavailability and high cellular toxicity at the effective concentrations, it is still necessary to seek alternative molecules disrupting the pri-miR-7/HuR/MSI2 complex. On the other hand, pre-treatment of OA exacerbated kidney fibrosis in unilateral ureteral obstruction (UUO) mouse models. This was related to increased P21 expression and enhanced Numb/Notch signalling as a result of MSI1 inhibition [160]. It implies that the MSI1-mediated pathways act as a double-edged sword in different human pathogenesis.

### 4.7. Other Molecules Identified from HTS Targeting MSI/RNA and Conclusions

(−)-gossypol is a natural product extracted from cottonseed. The molecule targets anti-apoptotic activities of Bcl-2 family proteins and has been studied in clinical trials for the treatment of a variety of cancers [153]. (−)-gossypol was identified as an MSI inhibitor in an FP-HTS carried out using GST tagged full-length MSI1, and fluorescein-labelled RNA probe (UAGGUAGUAGUUUUA), a known MSI1 binding motif located at the 3’-UTR of Numb mRNA [161]. The screen included about 2000 compounds from NCI (Diversity Set II) and an in-house library. (−)-gossypol showed 86.7% inhibition in the FP screen and a Ki of 476 ± 273 nM. The MSI inhibitor interacted with RRM1, as shown by SPR and NMR data [161]. Further in vitro and in vivo studies showed that (−)-gossypol could repress colon cancer growth through the control of downstream targets of MSI1 [161]. The discovery of the new target and pathway of (−)-gossypol provides an alternative route for its development as an anti-cancer drug. In a more recent FP-based screening, gossyopolone (Gn) was identified with a Ki of 12 ± 2 nM against full-length MSI1, and 7.0 ± 0.3 nM against full-length MSI2 [162]. Gn is a major metabolite of gossypol, and it showed higher affinity than (−)-gossypol in the same experimental conditions [162]. Gn disrupted the interaction between the Numb RNA probe and MSI1/MSI2 in the FP assay, displaying its role as a dual inhibitor [162]. The MSI inhibitor also directly interacted with the RRM1 of MSI1. Gn induced apoptosis and autophagy in colon cancer cells, with concomitant repression of Notch/Wnt signalling [162]. A Gn-loaded liposome (Gn-lip) was delivered to colon cancer xenograft mouse models through a tail vein injection. The treatment of Gn-lip inhibited tumour growth and downregulated Notch/Wnt downstream proteins in tumours [162]. The use of PEGylated liposomes improved the biocompatibility and drug efficacy of Gn in vivo, providing a strategy for drug delivery of MSI inhibitors with poor bioavailability.

An MSI1 inhibitor, luteolin, was discovered from an FP screening using a total of 25,588 compounds from several libraries, including Prestwick, LOPAC, Cambridge NovaCORE, and the Life Chemical FSP3-enriched libraries [163]. The assay utilised MSI1-RRM1, and 5’-Cy3 labelled RNA probe (GUAGUAGU) with a Z’ factor of 0.72. Luteolin directly binds to MSI1-RRM1 with a Kd of 3 μM, as determined by SPR. In GBM cells, the compound inhibited the expression levels of MSI1 targets including PDGFRα, IGF-IR, EGFR, CCND1, and CDK6. Importantly, it could suppress the MSI1-induced luminescence from the PDGFRα 3’-UTR luciferase reporter [163]. Moreover, luteolin inhibited proliferative and metastatic properties in GBM cells and organoids [163]. Surprisingly, the aforementioned HuR/TNF-α mRNA inhibitor quercetin is a close analogue of luteolin [134]. Quercetin also interacted with MSI1-RRM1 in the FP competition assay and presented similar but less potent anti-proliferation effects in GBM as luteolin [163]. This implies quercetin may act as a dual inhibitor targeting both HuR and MSI1 and interrupting their RNA-binding abilities. It would be interesting to see if quercetin also targets MSI2. If this were the case, it will become a promising candidate to rescue the HuR/MSI2-mediated inhibition on miR-7 biogenesis. Moreover, both quercetin and luteolin dissociated Lin28A/let-7g in vitro with IC50s less than 2.5 μM [55]. Despite the fact that these two flavone analogues appear to be less selective, they are interesting ligands targeting different RNA-binding domains and would be useful for the investigation of various RBP/RNA interactions.

So far, structural knowledge of HuR and Musashi proteins is very limited. The structures of HuR RRM1-2 and RRM3 were solved separately [123,124,164]. Similarly, MSI1 has two separate RRMs structures [165,166]. MIS2 only has RRM1 structures published [158,167]. Most of the HuR/ARE disruptors target the RRM1-2, while the sole RRM3 ligand has shown weak interference with full-length HuR/ARE complex [122]. How RRM3 coordinates with the N-terminal RRMs is yet to be completely understood. For Musashi proteins, all the above inhibitors interact with RRM1. The binding modes of the molecules with full-length Musashi proteins are unknown. Based on a model structure of RNA-bound MSI1 RRM 1–2, it has been deduced that the tandem RRMs will adopt certain orientations upon RNA binding [166]. Thereby, more information is needed to address the working mechanism of these drug candidates, in the context of full-length RBPs. Moreover, the interaction between pri-miR-7, HuR, and MSI2 has not been studied yet. Currently, our group is developing a novel screening approach targeting this pathway. Utilising the CONA platform, the screening will be performed in cell lysates, which may generate more bio-functionally relevant hits.

## 5. Other Discoveries Towards RNA-Targeted Therapies

Aside from the above three stories, there are more potential RNA-targeted therapies being developed. The following section will briefly outline some notable examples.

Myotonic dystrophy type 1 (DM1) is caused by the expansion of a (CTG)n repeat sequence in the dystrophia myotonica protein kinase (DMPK) gene 3’UTR [168]. From an in vitro HTS using ASOs complementary to DMPK mRNA, the ASO ISIS 486178 was described as the best candidate, exhibiting high potency in DMPK RNA reduction, as well as good tolerability in animals [169]. The lead ASO could ameliorate muscle weakness symptoms in DM1 mouse models, without exerting observed toxicity [170]. This provides evidence suggesting that a reduction of the levels of CUG RNA may improve the muscular function of DM1 patients [170].

A different expansion of G_4_C_2_ repeats in the c9orf72 gene is the most causative factor of amyotrophic lateral sclerosis (ALS) and frontotemporal dementia (FTD). The transcribed G_4_C_2_ repeats are responsible for the accumulation of toxic RNA foci, sequestration of proteins, as well as the production of dipeptide repeat proteins (DPRs), which are a result of non-ATG (RAN) translation [171]. From a FRET-based screen, small molecules targeting the G_4_C_2_ repeat RNA G-quadruplexes were identified. These RNA binders could reduce the G_4_C_2_ foci and DPRs in c9orf72 patient-derived neurons [172]. Another small molecule specifically binding the hairpin form of G_4_C_2_ repeat RNA was discovered from a fluorescence displacement assay. According to in vitro and cell-based assays, this molecule inhibits RAN translation and hnRNP-H sequestration of the target, blocking these two potential mechanisms driving the c9ALS/FTD pathogenesis [173].

In another study, a small molecule microarray (SMM) containing 20,000 drug-like molecules was previously amassed. SMM screening was performed against a fluorescently labelled RNA element, the HIV-1 transactivation response (TAR). This resulted in the discovery of TAR hairpin stabilisers, which provide anti-HIV activity in T-lymphocytes [174]. Further work was performed to uncover the noncanonical binding mode between TAR and the synthesised derivatives of the initial hit compound [175].

The discovery of small-molecule miRNA regulators has been previously reviewed, summarising three major strategies, including HTS from large libraries, focused screening using known RNA binders, such as aminoglycosides, as well as drug design targeting RNA secondary structures or relevant RBPs [176]. The use of focused screening leads to a higher success rate than HTS; however, the resultant RNA ligands are likely to lack selectivity. As for the drug design, it is necessary for it to be based on previously validated, functional miRNA modulators [176].

Streptomycin is a well-known aminoglycoside antibiotic. It was previously identified as a miR-21 inhibitor, from a focused screen using a cell-based luciferase reporter assay [177]. Streptomycin binds to pre-miR-21, and thus blocks Dicer cleavage. Reduced miR-21 levels allow the rescue of its targets, such as PDCD4. This could translate into the potential for cancer therapy, with the restored PDCD4 function observed after streptomycin treatment in vitro and in cells [177].

Finally, Disney et al. developed a novel focused screen platform named dubbed two-dimensional combinatorial screening (2DCS). This is a library-vs-library screen where a small molecule microarray is hybridised with an RNA element library, allowing the definition of interactions between the compounds and steric RNA structures [178,179]. Using a 2DCS against two aminoglycosides derivatives, the internal loop of pri-miR-10 was found to bind G Neo B, which prevents Drosha processing during miR-10 biogenesis [180]. More recently, the Disney group identified a functional inhibitor of the oncogenic miR-18a, based on 2DCS combined with statistical analysis approaches [181].

These RNA binders have shown great potential as future therapeutics, targeting viral RNA, oncogenic miRNA, as well as abnormal mRNA repeats. To find out whether and how the relevant RBPs become involved in the field will be a topic of great interest.

## 6. Conclusions and Future Perspectives

Throughout this review, we have summarised commercialised RNA-targeted therapies and those under evaluation in clinical and preclinical studies. These drugs are based on different formats including ASOs, siRNAs, miRNA mimics and small molecules. The therapeutic effects are achieved through the regulation of RNA maturation, splicing, interference, translation and stabilisation. More possibilities of drug formats and therapeutic mechanisms have been discussed, including peptides [182], aptamers [183] and RNA editing with CRISPR-Cas13 [184]. Over the past five years, an increasing number of RNA-targeted drugs have been approved. There is no doubt that more will be utilised to enable the treatment of previously “incurable” diseases in the near future. Spinraza, the first effective SMA drug, is an excellent example.

By reviewing a number of HTS targeting RBPs and their regulatory RNAs, we have reached several important conclusions, which may benefit strategy making for future RNA-targeted drug discovery in the preclinical stage. First of all, we have shown a collection of different screening assays. FRET and FP are the most common assays applied in the identification of disruptors between two-component RBP-RNA interactions. Luciferase minigene reporter assays have been frequently used to identify splicing modifies. This is a paradigm of phenotypic screens. Alternatively, some novel screening strategies are being developed, such as CONA and FD assay [107,122]. More RNA-targeted HTS approaches have been reported, such as in silico virtual screens, and small molecule microarray screening [185,186]. Secondly, the understanding of RNA motifs and RBP domains in the interaction interface is crucial. This is true for the selection of RNA probes, tagging of the protein domains, as well as for enabling the study of the mechanism of candidate drugs. Sometimes, the discovery of ligands can, in turn, elucidate the action mechanism of RBP-RNA interplays. For example, an ATP-binding pocket was identified by studying the inhibitors of HuR-RRM3 [122]. Cis-elements controlling SMN2 pre-mRNA splicing were confirmed through ASO screenings [77,106]. Finally, positive hits obtained from screening assays need to be validated in more physiologically relevant models. Ideally, hits should be tested against full-length RBPs, as well as being investigated in the cellular context to determine whether they can still regulate the metabolism of the target RNAs.

Several aforementioned hit compounds have shown promising therapeutic effects in the preclinical and clinical tests. Risdiplam is the most advanced investigational medicine, awaiting the FDA’s decision under priority review. It is being studied in all types of SMA patients aged from 0-60 years old. If approved, risdiplam will become the first self-administrated SMA medicine. Some of the Lin28/let-7 disruptors (1632 and KCB3602), as well as HuR (MS-444, DHTS and CMLD-2) or MSI inhibitors (Ro, luteolin, (−)-gossypol and Gn), have exhibited anti-cancer properties in vitro or in vivo. However, further research needs to be undertaken to evaluate their efficacy, bioavailability and safety before entering the clinical studies. Beyond these three examples, therapies targeting viral RNAs [187,188], RNA cleavage through RNase recruitment [189,190], mRNA translation by inhibiting eukaryotic translation initiation factors [191], as well as premature translational termination caused by nonsense mutations [192], are being developed. With respect to the huge number of RNA-targeted therapies in preclinical studies and clinical trials, it is apparent that these therapies will form a key part of personalised, precision medicine in the future.

## Figures and Tables

**Table 1:** Marketed RNA-targeted therapies.

Drug	Disease	Target RNA	Therapy Type	Company	Ref.	Year Approved
Exondys 51/Eteplirsen	Duchenne’s muscular dystrophy (DMD)	Dystrophin exon 51	ASO	Sarepta Therapeutics	[14]	2016 (FDA)
Givlaari™/Givosiran	Acute hepatic porphyria	ALAS1	RNAi	Alnylam Pharmaceuticals	[15]	2019 (FDA)
Kynamro^®^/Mipomersen	Hypercholesterolaemia	ApoB-100	ASO	Ionis Pharmaceuticals/Kastle Therapeutics	[16]	2013 (FDA)
Onpattro^®^/Patisiran	Hereditary TTR amyloidosis	TTR	RNAi	Alnylam Pharmaceuticals	[17]	2018 (FDA)
Ribavirin	RSV infection /Hepatitis C/viral haemorrhagic fevers	Viral RNA	Small molecule	Multi companies		2002 (FDA)
Spinraza^®^/Nusinersen	Spinal muscular atrophy (SMA)	SMN2	ASO	Ionis Pharmaceuticals/ Biogen	[18]	2016 (FDA)
Tegsedi^®^/Inotersen	Hereditary TTR amyloidosis	TTR	ASO	Ionis Pharmaceuticals/ Akcea Therapeutics	[19]	2018 (FDA)
Translarna^®^/Ataluren	DMD	Dystrophin	Small molecule	PTC Therapeutics	[20]	2014 (EMA)
Vitravene/Fomivirsen	CMV retinitis in HIV patients	Major intermediate early region 2 of CMV	ASO	Ionis Pharmaceuticals/ Novartis	[21]	1998 (FDA)
Vyondys 53/Golodirsen	DMD	Dystrophin exon 53	ASO	Sarepta Therapeutics	[22]	2019 (FDA)
Waylivra^®^/Volanesorsen	Hypertriglyceridaemia/Lipodystrophy	ApoC-III	ASO	Ionis Pharmaceuticals/ Akcea Therapeutics	[23]	2019 (EMA)

**Table 2:** Representative RNA-targeted therapies in clinical trials.

Drug	Disease	Target RNA or RBP *	Therapy Type	Phase	Trial #
ALN-AT-3/Fitusiran	Haemophilia A/B	AT3	RNAi	III	NCT03549871 NCT03754790 NCT03417102 NCT03417245
ALN-GO1/Lumasiran	Primary hyperoxaluria type I	HAO1	RNAi	III	NCT04152200 NCT03905694 NCT03681184
ALN-PCSSC/Inclisiran	Hypercholesterolaemia	PCSK9	RNAi	III	NCT03814187 NCT03399370 NCT03397121 NCT03705234
Branaplam/LMI070	SMA	SMN2	Small molecule	I/II	NCT02268552
ISIS 2302/AP 1007/Alicaforsen	Pouchitis/Crohn’s Disease	ICAM1	ASO	III	NCT02525523 NCT00048113
LY900003/ISIS 3521	Non-small cell lung carcinoma	PKC-*a*	ASO	III	NCT00017407 NCT00034268
MesomiR-1	malignant pleural mesothelioma/non-small cell lung cancer	miR-16	miRNA mimic	I	NCT02369198
MRG-106/Cobomarsen	Cutaneous T-cell lymphoma/Mycosis fungoides	miR-155	ASO	I/II	NCT02580552 NCT03713320 NCT03837457
MRG-201/MiR-29/ Remlarsen	Keloid	miR-29b	miRNA mimic	II	NCT03601052
OGX-011/Custirsen	Cancer	Clusterin	ASO	III	NCT01188187 NCT01578655
RG-012/SAR339375	Alport’s syndrome	miR-21	ASO	II	NCT02855268
RG6042/IONIS-HTT _RX_/ISIS 443139	Huntington’s disease	HTT	ASO	III	NCT03842969 NCT03761849
RG7916/RO7034067/Risdiplam	SMA	SMN2	Small molecule	II/III	NCT03779334 NCT02913482 NCT03032172 NCT02908685
RPI.4610/Angiozyme	Kidney cancer	VEGFR-1	Ribozyme	II	NCT00021021
SPC3649/Miravirsen	Chronic Hepatitis C	miR-122	ASO	II	NCT02508090 NCT02452814 NCT01200420
Zotatifin /EFT226	Solid tumour	EIF4A1 *	Small molecule	I/II	NCT04092673

* If the target is an RBP. #—Number.

## Supplementary Materials

Supplementary materials can be found at https://www.mdpi.com/1422-0067/21/8/2996/s1. Supplementary Table S1: RNA-targeted therapies in clinical trials.

## Abbreviations

2AP	2-aminopurine
2D-FIDA	two-dimensional fluorescence intensity distribution analysis
ALAS1	5’-aminolevulinate synthase 1
ApoA/B/C	apolipoprotein A/B/C
ARE	AU-rich element
ASO	antisense oligonucleotide
AT3	antithrombin III
ATP	adenosine triphosphate
Bcl-2	B-cell lymphoma 2
BHQ	black-hole-quencher
*C.elegans*	Caenorhabditis elegans
cat-ELCCA	catalytic enzyme-linked click chemistry assay
CMV	cytomegalovirus
CONA	confocal nanoscanning
CRISPR-Cas	clustered regularly interspaced short palindromic repeats-associated protein
CSD	cold-shock domain
DGCR8	DiGeorge syndrome critical region 8
DHTS	15,16-dihydrotanshinone-I
DMD	Duchenne’s muscular dystrophy
EC50	The half-maximal effective concentration
EGFR	epidermal growth factor receptor
EIF4A	eukaryotic translation initiation factor 4A
ELISA	enzyme-linked immunosorbent assay
EMSA	electrophoretic mobility shift assay
ESC	embryonic stem cells
ESE	exonic splicing enhancer
ESS	exonic splicing silencer
FD assay	fluorescence displacement assay
FDA	Food and Drug Administration
FL assay	fluorescence intensity-based binding assay
FP	fluorescence polarisation
FRET	fluorescence resonance energy transfer
GBM	glioblastoma
Gn	gossyopolone
GST	glutathione S-transferase
HAO1	hydroxyacid oxidase 1
HIV	human immunodeficiency virus
HMGA2	high mobility group AT-hook 2
hnRNP	heterogeneous nuclear ribonucleoprotein
HPLC	high-performance liquid chromatography
HTS	high-throughput screening(s)
HTT	huntingtin
HuR	human antigen R
IC50	the half-maximal inhibitory concentration
ICAM1	intercellular adhesion molecule 1
IL	interleukin-2
IP	immunoprecipitation
IR	infrared spectroscopy
ISE	intronic splicing enhancer
ISS	intronic splicing silencer
Kd	dissociation constant
Ki	inhibition constant
KRAS	Kirsten rat sarcoma 2 viral oncogene homolog
Lin28A/B	protein lin-28 homolog A/B
miR/miRNA	microRNA
mRISC	miRNA-induced silencing complex
mRNA	messenger RNA
MS	mass spectrometry
MSI	Musashi RNA binding protein
MST	MicroScale Thermophoresis
HRP	horseradish peroxidase
MUFA	monounsaturated fatty acid
MYC	MYC proto-oncogene
NCI	National Cancer Institute
NMR	nuclear magnetic resonance
nt	nucleotides
OA	oleic acid
PCSK9	proprotein convertase subtilisin/kexin type 9
PD	Parkinson’s disease
PKC	protein kinase C
PK4C9	homocarbonyltopsentin
PLK	polo-like kinase
pre-miR	precursor microRNA
pre-mRNA	precursor messenger RNA
pri-miR	primary microRNA
qRT-PCR	quantitative reverse transcription-polymerase chain reaction
RBP	RNA-binding protein
RNA	ribonucleic acid
RNAi	RNA interference
RRM	RNA recognition motif
RSV	respiratory syncytial virus
SAR	Structure-activity relationship
siRNA	small interfering RNA
SMA	spinal muscular atrophy
SMN	survival motor neuron
snRNP	small nuclear ribonucleoproteins
SOX2	sex-determining region y-box2
SPR	surface plasma resonance
SR proteins	serine-arginine-rich proteins
SRSF2	serine/arginine rich splicing factor 2
ss	splicing site
SYNCRIP	synaptotagmin binding cytoplasmic RNA interacting protein
TNF-α	tumour necrosis factor-α
Trim25	tripartite motif-containing 25
TSL2	terminal stem-loop 2
TTR	transthyretin
TUT4	terminal uridylyltransferase 4
UTR	untranslated region
VEGFR	vascular endothelial growth factor receptor
XIAP	X-linked inhibitor of apoptosis protein
