# Supplementary material for: RNA-Targeted Therapies and High-Throughput Screening Methods
Source: Int J Mol Sci. 2020 Apr 23;21(8):2996. doi: 10.3390/ijms21082996 (PMC7216119; doi:10.3390/ijms21082996)
Supplement: Supplementary file 1 [file ijms-21-02996-s001.pdf]

Supplementary Table: RNA-targeted therapies in clinical trials.

Drug	Disease	Target RNA or RBP*	Therapy Type	Phase	Reference	Notes
AEG35156	Cancer	XIAP	ASO	II	NCT00363974<br>NCT00882869	In combination with chemotherapies / Multiple studies terminated
AGN 211745 / Sirna-027	Choroid neovascularisation / age-related macular degeneration	VEGFR1	RNAi	II	NCT00395057	Terminated—company decision
AKCEA-TTR-LRx	hATTR-Amyloid Cardiomyopathy /hATTR-amyloid polyneuropathy	TTR	ASO	III	NCT04136171<br>NCT04136184	
ALN-AAT02	Alpha-1 liver disease	Alpha-1 antitrypsin (AAT)	RNAi	I/II	NCT03767829	
ALN-AGT	Hypertension / pre-eclampsia	Angiotensinogen (AGT)	RNAi	I	NCT03934307	
ALN-AT-3 / Fitusiran	Haemophilia A/B	Anti-thrombin III (AT3, SERPINC1)	RNAi	III	NCT03549871<br>NCT03754790<br>NCT03417102<br>NCT03417245	
ALN-CC5 / Cemdisiran	Complement mediated diseases	C5 complement	RNAi	II	NCT03841448<br>NCT03999840	
ALN-GO1 / Lumasiran	Primary hyperoxaluria type I	HAO1	RNAi	III	NCT04152200<br>NCT03905694<br>NCT03681184	
ALN-HBV02 / VIR2218	Chronic HBV	HBV RNA	RNAi	I/II	NCT03672188	
ALN-PCSSC / Inclisiran	Hypercholesterolemia	PCSK9	RNAi	III	NCT03814187<br>NCT03399370<br>NCT03397121<br>NCT03705234	In the process of registration
ALN-TTRsc / Revusiran	hATTR amyloidosis / Familial amyloidotic cardiomyopathy	TTR	RNAi	III	NCT02319005	Terminated due to deaths
ALN-TTRsc02 / Vutrisiran	hATTR amyloidosis	TTR	RNAi	III	NCT04153149<br>NCT03759379	
ALN-VSP02	Solid tumours	VEGF / KSP	RNAi	I	NCT00882180<br>NCT01158079	
AMG 890 / ARO-LPA / ARC-LPA	Cardiovascular disease	ApoA	RNAi	I/II	NCT03626662<br>NCT04270760	
APN 401	Solid tumours	CBL-B	RNAi	I	NCT03087591<br>NCT02166255	
ARC-AAT	Alpha-1 antitrypsin deficiency	AAT	RNAi	I/II	NCT02900183<br>NCT02363946	Terminated / withdrawn – company decision
ARO-AAT	Alpha-1 antitrypsin deficiency	Z-AAT	RNAi	I / II / III	NCT03362242<br>NCT03946449<br>NCT03945292	
ARO-ANG3	Dyslipidemia	Angiopoietin-like protein 3 (ANGPTL3)	RNAi	I	NCT03747224	
ARO-APOC3	Hypertriglyceridaemia	ApoC-III	RNAi	I	NCT03783377	
ARO-HIF2	Clear cell renal carcinoma	HIF-2α	RNAi	I	NCT04169711	
ARO-HSD	Non-alcoholic steatohepatitis	HSD17B13	RNAi	I	NCT04202354	
ARO-HBV / JNJ-3989	Hepatitis B	HBV mRNA	RNAi	I	NCT04208386	
ATU027	Solid tumours/ advanced pancreatic cancer	Protein kinase N3 (PKN3)	RNAi	I/II	NCT00938574<br>NCT01808638	Combined with gemcitabine
AVI-7100 / Radavirsen	Influenza	Influenza M1/M2	ASO	I	NCT01747148	

AZD4076 / RG-125	NASH with TII diabetes / pre-diabetes	Mir-103/107	ASO(s)	I/II	NCT02612662<br>NCT02826525	
AZD4785 / IONIS-KRAS-2.5Rx	Advanced solid tumours	KRAS	ASO	I	NCT03101839	
AZD5312 / IONIS-AR-2.5RX / ARRx	Cancer – prostatic	Androgen receptor (AR)	ASO	I/II	NCT03300505	
BMS-986263 / ND-L02-S0201	Hepatic impairment / NASH / Cirrhosis / Fibrosis	HSP47	RNAi	I/II	NCT04225936<br>NCT04267393<br>NCT03420768	
BP1001 / Prexigebersen	Cancer	GRB-2	ASO	I/II	NCT04196257<br>NCT02923986<br>NCT02781883<br>NCT01159028	In combination with chemotherapies
BP1002	Advanced lymphoid malignancy	Bcl-2	ASO	I	NCT04072458	
Branaplam/ LMI070	SMA	SMN2	Small molecule	I/II	NCT02268552	
Briciclib /ON 013105	Advanced solid tumour	eIF4E*	Small molecule	I	NCT02168725	Suspended
CALAA-01	Solid tumours	M2 subunit of ribonucleotide reductase (R2)	RNAi	I	NCT00689065	Terminated
Cand5 / Bevasiranib	Macular degeneration	VEGF	RNAi	III	NCT00557791<br>NCT00499590	Withdrawn / Terminated
DCR-A1AT	Alpha-1 anti-trypsin deficiency	A1AT	RNAi	I/II	NCT04174118	
DCR-HBVS / RG6346	Chronic hepatitis B	HBV mRNA	RNAi	I	NCT03772249	
DCR-MYC	Solid tumours	MYC	RNAi	I/II	NCT02314052	Terminated – sponsor decision
DCR-PH1	Primary hyperoxaluria type I	HAO1	RNAi	I	NCT02795325	Terminated
DCR-PHXC / Nedosiran	Primary hyperoxaluria type	LDHA	RNAi	II / III	NCT03847909<br>NCT04042402	
DS-5141b	DMD	Distrophin -exon 45	ASO	I/II	NCT02667483	
EL625 / Cenersen / Aezea®	Myelodysplastic syndromes / Leukaemia / Lymphoma	P53	ASO	II	NCT00074737	Other phase II terminated due to lack of funding
EphA2-targeting DOPC-encapsulated siRNA	Advanced malignant solid neoplasm	EphA2	RNAi	I	NCT01591356	
EZN-2968 / Anti-HIF-1*a* / LNA AS ODN	Solid tumour	HIF-1*a*	ASO	I	NCT01120288<br>NCT00466583<br>NCT02564614	
EZN-4176	Prostatic neoplasm	AR exon 4	ASO	I	NCT01337518	Suspended
G3139 / Oblimersen / Genasense®	Cancer / Waldenstroms Macroglobulinaemia	Bcl-2	ASO	II/III	NCT01200342<br>NCT00543205	Terminated studies due to discontinued development
G4460 / C-MYB asODN	Haematological malignancy	C-Myb	ASO	II	NCT00002592	
GRN163L / Imetelstat®	Cancer	RNA component of telomerase	ASO	II/III	NCT02598661	
GS-101 / Aganirsen	Ischaemic central retinal vein occlusion / neovascular glaucoma	Insulin receptor substrate-1 (IRS-1)	ASO	II/III	NCT02947867	Unknown status
GSK2402968 / Drisapersen	DMD	Distrophin -exon 51	ASO	III	NCT01254019	
GSK 299872 / ISIS-420915	Amyloidosis	TTR	ASO	II	NCT02627820	Withdrawn
GTI-2040	Cancer	R2 component of R2	ASO	II	NCT00565058<br>NCT00068588<br>NCT00087165	In combination with chemotherapies
GTX-102	Angelmann's syndrome	UBE3A	ASO	I/II	NCT04259281	

IGF-1R/AS ODN	Glioblastoma	IGF-1R	ASO	I	NCT02507583<br>NCT01550523	
ION839 / AZD2693 / IONIS-AZ6-2.5-LRX	NASH	PNPLA3	ASO	I	NCT04142424	
ION859 / BIIB094	Parkinson's disease	LLRK2	ASO	I	NCT03976349	
IONIS-AGT-LRX	Hypertension	AGT	ASO	II	NCT04083222<br>NCT03714776	
IONIS-ANGPTL3-LRX / AKCEA-ANGPTL3-LRX / ISIS 703802 / Vupanorsen	Dyslipidaemias / NAFLD / TII diabetes	ANGPTL3	ASO	II	NCT03371355<br>NCT03360747<br>NCT02709850<br>NCT03514420	
IONIS-APOCIII-LRX / AKCEA-APOCIII-LRX / ISIS 678354	Hypertriglyceridemia / cardiovascular disease	ApoC-III	ASO	II	NCT03385239	
IONIS-C9RX / BIIB078	Amyotrophic lateral sclerosis	C9orf72	ASO	I	NCT04288856<br>NCT03626012	
IONIS-DGAT2RX	Hepatic steatosis / NASH	DGAT2	ASO	II	NCT03334214	
IONIS-ENaCRX	Cystic fibrosis	ENaC	ASO	I	NCT03647228	
IONIS-FB-LRX	Complement-mediated diseases	Complement factor B (CFB)	ASO	II	NCT04014335<br>NCT03815825	
IONIS-FGFR4RX / ISIS-FGFR4RX	Obesity	FGFR4	ASO	II	NCT02476019	
IONIS-FXIRX / ISIS 416858 / BAY2306001	Clotting disorders	FXI	ASO	II	NCT02553889<br>NCT03358030<br>NCT01713361	
IONIS-GCGRRX / ISIS-GCGRRX	TII Diabetes	GCGR	ASO	II	NCT02583919<br>NCT02824003<br>NCT01885260	
IONIS-GHR-LRX	Acromegaly	GHR	ASO	II	NCT03967249<br>NCT03548415	
IONIS-HBV-LRX / GSK3389404	Chronic Hep B	HBV mRNA	ASO	II	NCT03020745	
IONIS-HTTRX / ISIS 443139 / RG6042	Huntington's disease	HTT	ASO	III	NCT03842969<br>NCT03761849	
IONIS-MAPTRX / BIIB080	Mild Alzheimer's disease	Tau	ASO	I/II	NCT03186989	
IONIS-PKK-LRX	Chronic migraine / hereditary angioedema	Prekallikrein (PKK)	ASO	II	NCT04030598<br>NCT04307381	
IONIS-SOD1RX / BIIB067 / Tofersen	Amyotrophic lateral sclerosis	SOD1	ASO	III	NCT02623699	
IONIS-STAT3RX / AZD9150 / Danvatirsen	Cancer	STAT3	ASO	I/II	NCT01563302	
IONIS-TMPRSS6-LRX	Beta Thalassaemia	TMPRSS6	ASO	II	NCT04059406	
ISIS 104838	Rheumatoid arthritis	TNF-α	ASO	II	NCT00048321	
ISIS 113715	TII diabetes	PTP-1B	ASO	II	NCT00455598<br>NCT00330330	
ISIS 2302 / AP 1007 / Alicaforsen	Pouchitis/ Crohn's Disease	ICAM1	ASO	III	NCT02525523<br>NCT00048113<br>NCT00048295	In process of registration

ISIS 5132 / CGP69846A	Ovarian / breast cancer	C-Raf-1	ASO	II	NCT00003236<br>NCT00003892	
ISIS-CRP$_{RX}$ / ISIS 353512	Atrial fibrillation / rheumatoid arthritis	CRP	ASO	II	NCT01710852<br>NCT01414101	
ISIS-PTP1B$_{RX}$	TII diabetes	PTP-1B	ASO	II	NCT01918865	
ISTH0036	Open-angle glaucoma	TGF-β2	ASO	I	NCT02406833	
LErafAON-ETU	Cancer	C-Raf-1	ASO	I	NCT00100672	
LY900003 / ISIS 3521	Cancer	PKC-α	ASO	III	NCT00017407<br>NCT00034268	In combination with chemotherapies
MesomiR-1	Malignant pleural mesothelioma / non-small cell lung cancer	miR-16	miRNA mimic	I	NCT02369198	
MRG-106 / Cobomarsen	Cutaneous T-cell lymphoma / Mycosis fungoides	miR-155	ASO	I/II	NCT02580552<br>NCT03713320<br>NCT03837457	
MRG-110 / S95010	Wounds	miR-92a	ASO	I	NCT03603431	
MRG-201 / MiR-29/ Remlarsen	Keloid	miR-29b	miRNA mimic	II	NCT03601052	
MRX34	Cancer	miR-34	miRNA mimic	I/II	NCT01829971<br>NCT02862145	Terminated<br>Withdrawn
OGX-011 / Custirsen	Cancer	Clusterin	ASO	III	NCT01188187<br>NCT01578655	In combination with chemotherapies
OGX-427 / Apatorsen	Cancer	HSP27	ASO	II	NCT01120470<br>NCT02423590<br>NCT01829113<br>NCT01454089	In combination with chemotherapies
OLX10010	Hypertrophic cicatrix	CTGF	RNAi	I	NCT03569267	
QPI-1002 / I5NP	Delayed graft function in kidneys / other adverse renal events	P53	RNAi	III	NCT03510897<br>NCT02610296	
QPI-1007	Non-arteritic ischemic optic neuropathy / other optic neuropathies	Caspase 2	RNAi	II/III	NCT02341560	Terminated due to interim analysis
QR-010 / Eluforsen	Cystic fibrosis	CFTR	ASO	I/II	NCT02532764	
QR-110 / Sepofarsen	Leber congenital amaurosis	CEP290	ASO	II/III	NCT03913143	
QR-1123 / ION357	Autosomal dominantretinitis pigmentosa	Rhodopsin	ASO	I/II	NCT04123626	
QR-313	Recessive epidermolyisis bullosa dystrophica	COL7A1 exon 71	ASO	I/II	NCT03605069	
QR-421a	Usher syndrome	USH2A exon 13	ASO	I/II	NCT03780257	
PF-04523655 / PF-655 / REDD14NP	Diabetic macular oedema / age-related macular degeneration	RTP801	RNAi	II	NCT01445899	
PRO-040201	Hypercholesterolaemia	ApoB	RNAi	I	NCT00927459	Terminated—potential immune stimulation
RG-012 / SAR339375	Alport's syndrome	miR-21	ASO	II	NCT02855268	
RG6042 / IONIS-HTT$_{RX}$ / ISIS 443139	Huntington's disease	HTT	ASO	III	NCT03842969<br>NCT03761849	
RG7916 / RO7034067 / Risdiplam	SMA	SMN2	Small molecule	II/III	NCT03779334<br>NCT02913482<br>NCT03032172<br>NCT02908685	Registration in progress
RO7070179	Hepatocellular carcinoma	HIF-1α	ASO	I	NCT02564614	
RPI.4610 / Angiozyme	Kidney cancer	VEGFR-1	Ribozyme	II	NCT00021021	
SB010	Asthma	GATA-3	ASO	II	NCT01743768	
SB011	Atopic dermatitis	GATA-3	ASO	II	NCT02079688	
SB012	Ulcerative colitis	GATA-3	ASO	I/II	NCT02129439	

SiG12D LODER	Pancreatic cancer / pancreatic ductal carcinoma	KRAS G12D	RNAi	II	NCT01676259	In combination with chemotherapy
SLN124	Beta-thalassaemia / myelodysplastic syndrome	TMPRSS6	RNAi	I	NCT04176653	
SPC2996	Chronic lymphocytic leukaemia	Bcl-2	ASO	I/II	NCT00285103	
SPC3649 / Miravirsen	Chronic Hepatitis C	miR-122	ASO	II	NCT02508090 NCT02452814 NCT01200420	
SPC4955	Hypercholesterolaemia	ApoB	ASO	I	NCT01365663	
SRP-4045 / Casimersen	DMD	Distrophin-exon 45	ASO	II / III	NCT04179409 NCT03532542	
SPC5001	Hypercholesterolaemia	PCSK9	ASO	I	NCT01350960	
SRP-5051	DMD	Distrophin-exon 51	ASO	I/II	NCT03675126 NCT04004065	
STP705 / Cutasil	Bowen's disease / Cutaneous squamous cell carcinoma / hypertrophic scar	TGF-β1 / COX2	RNAi	I/II	NCT04293679 NCT02956317	
SXL01	Prostatic cancer	AR	RNAi	I	NCT02866916	
SYL040012 / Bamosiran	Open-angle glaucoma / ocular hypertension	ADRB2	RNAi	II	NCT02250612	
SYL1001 / Tivanisiran	Dry eye disease	TRPV1	RNAi	III	NCT03108664	
TD101	Pachyonychia congenita	Keratin 6a	RNAi	I	NCT00716014	
TKM080301	Cancer	PLK1	RNAi	I/II	NCT02191878 NCT01262235	
TKM130803/Tekmira	Ebola Virus Disease	Ebola virus RNA	RNAi	II	PACTR201501000997429	
TPI ASM8	Asthma	IL-3 / IL-5 / GM-CSF / CCR3	ASOs	II	NCT00550797 NCT01158898 NCT00822861	
TQJ230 / IONIS-APO-L$_{RX}$ / ISIS 681257 / AKCEA-APO-L$_{RX}$	Cardiovascular disease	Lp(a)	ASO	III	NCT04023552	
TT-034	Hepatitis C	HCV mRNA	RNAi	I/II	NCT01899092	
VEGF-AS / Veglin TM	Mesothelioma	VEGF	ASO	I/II	NCT00668499	Withdrawn—sponsor withdrew support
WVE-120101	Huntington's disease	HTT	ASO	I/II	NCT03225833	
WVE-120102	Huntington's disease	HTT	ASO	I/II	NCT03225846	
WVE-210201/ Suvodirsen	DMD	Distrophin exon 51	ASO	II/III	NCT03907072	Terminated—lack of efficacy
Zotatifin /EFT226	Solid tumour	EIF4A1*	Small molecule	I/II	NCT04092673	
